# Author’s Correction

**DOI:** 10.1264/jsme2.ME11126e

**Published:** 2012-06-09

***Minireview***

**Oxidative Stress Induced in Microorganisms by Zero-valent Iron Nanoparticles**

Alena Ševců^1^*, Yehia S. El-Temsah^2^, Erik J. Joner^2^, and Miroslav Černík^1^

*^1^Institute for Nanomaterials, Advanced Technologies and Innovations, Technical University of Liberec, Studentská 2, 461 17 Liberec, Czech Republic; and^2^Bioforsk Soil and Environment, Fredrik A Dahls vei 20, NO-1432 Ås, Norway*

Volume 26, no. 4, Page 271–281, 2011

Page 278, Acknowledgements, ‘This review was partially supported by the Czech Ministry of Education (1M0554) and by the Ministry of Industry and Trade (FR-TI3/564)’.

